# Mapping the knowledge domain: a bibliometric analysis of global research on traditional Chinese medicine for non-alcoholic fatty liver disease (2000–2024)

**DOI:** 10.3389/fmed.2026.1744929

**Published:** 2026-03-20

**Authors:** Zhongwen Feng, Qingliu Lu, Yinglian Wang, Xuefeng Jin, Yunyun Qin, Kefeng Li, Xiaoyu Chen

**Affiliations:** 1 Centre for Artificial Intelligence Driven Drug Discovery, Faculty of Applied Sciences, Macao Polytechnic University, Macau, Macao SAR, China; 2 Department of Pharmacy, Guangxi Academy of Medical Sciences and the People's Hospital of Guangxi Zhuang Autonomous Region, Nanning, China; 3 Pharmaceutical College, Guangxi Medical University, Nanning, China; 4 Department of Nursing, Guangxi Academy of Medical Sciences and the People’s Hospital of Guangxi Zhuang Autonomous Region, Nanning, China

**Keywords:** bibliometric analysis, bibliometrix, CiteSpace, non-alcoholic fatty liver disease, research hotspots, traditional Chinese medicine, VOSviewer

## Abstract

**Objective:**

Non-alcoholic fatty liver disease (NAFLD) constitutes a significant global health burden with rising prevalence. While Traditional Chinese Medicine (TCM) exhibits growing potential in NAFLD intervention, no domain-specific bibliometric evaluation currently exists. Utilising the most recent data from authoritative bibliographic databases, this study conducts a comprehensive bibliometric analysis to delineate the knowledge structure, research fronts, and collaborative networks in this field.

**Methods:**

We searched for publications from 2000 to 2024 in the Web of Science Core Collection (WoSCC) database, encompassing a total of 855 papers. In addition, a supplementary search was conducted in the PubMed database to identify and analyze eligible clinical trials. Bibliometric analyses were performed utilising R software, VOSviewer, and CiteSpace.

**Results:**

Investigations into TCM about NAFLD have indicated a general upward trajectory. China leads in research output, succeeded by the United States and South Korea. Shanghai University of Traditional Chinese Medicine is the preeminent cooperative institution. Ji G ranks as the most productive author in this field, whereas Younossi ZM emerges as the most frequently co-cited scholar. Among journals, Journal of Ethnopharmacology publishes the largest number of articles, while Hepatology receives the highest citation frequency. Key research themes include gut microbiota, network pharmacology, inflammation, insulin resistance, and lipid metabolism. Research hotspots primarily concentrate on the mechanisms by which TCM compounds, like berberine and Lingguizhugan Decoction, have garnered considerable attention, and the utilisation of contemporary research methodologies, such as network pharmacology, has markedly intensified.

**Conclusion:**

This bibliometric analysis thoroughly outlines the current status and developmental tendencies of TCM research in NAFLD for the first time, offering significant references for future investigations in this domain.

## 1 Introduction

Non-alcoholic fatty liver disease (NAFLD) refers to a spectrum of long-term liver conditions characterized by abnormal fat accumulation in hepatocytes, in the absence of significant alcohol intake or other hepatic etiologies. The pathophysiology is intricate, encompassing various pathological processes including lipid metabolic problems, insulin resistance, oxidative stress, chronic inflammation, and intestinal dysbiosis (1, 2). NAFLD encompasses a continuum from non-alcoholic fatty liver (NAFL), characterized by benign steatosis, to non-alcoholic steatohepatitis (NASH), which features hepatocellular injury, inflammatory infiltration, and ballooning degeneration. Without intervention, NASH can progress to advanced fibrosis, cirrhotic transformation, and in some cases, hepatocellular carcinoma (3). The worldwide surge in obesity and type 2 diabetes has contributed to the growing incidence of NAFLD across various populations. It is currently acknowledged as one of the most prevalent chronic liver disorders globally, impacting an estimated one-third of the world’s population (4, 5). In the last 30 years, mortality associated with NAFLD has significantly risen. In particular, regions such as Asia and the Middle East have seen substantial rises in NAFLD-related mortality (6). Despite its high burden, no approved pharmacological therapies exist, and current clinical management centers on dietary modification and exercise (7–9); however, patients have difficulty with long-term adherence, and overall efficacy is limited, so it is critical to find effective potential treatment options.

Traditional Chinese Medicine (TCM), originating in China as a foundational pillar of its cultural heritage and medical system, has extended its influence across East Asia while remaining predominantly practiced and developed within China. Its multi-component, multi-target regulatory paradigm has been widely applied in managing chronic complex disorders. As of now, some Chinese herbal remedies, including *Salvia miltiorrhiza* (10), *Pueraria lobata* (11), and Cassiae Semen (12), have been documented to provide advantageous effects on NAFLD. In addition, several classic TCM formulas, including Chaihu Shugan Powder (13) and Yinchenhao Decoction (14), have also demonstrated therapeutic potential through synergistic interactions among their constituent herbs. These herbal medicines and their active components show broad application prospects in the treatment of NAFLD by lipid metabolism regulation, exerting antioxidant and anti-inflammatory effects, preventing fibrosis, and modulating the gut microbiota (15, 16). In contrast to Western medicine, which typically relies on single-target synthetic agents, TCM adopts a holistic therapeutic approach based on Bian Zheng Lun Zhi (syndrome differentiation) (17). This conceptual framework provides innovative insights and approaches for the management of NAFLD.

Bibliometrics is a methodology that examines the quantitative attributes of the literary corpus and its publications through mathematical, statistical, and econometric techniques. It thoroughly elucidates the developmental tendencies and hotspot evolution of a certain research domain from several angles, including temporal, geographical, authorship, institutional, and keyword analyses (18, 19). Prevalent bibliometric instruments comprise VOSviewer (20), CiteSpace (21), and R-bibliometrix (22). In recent years, bibliometrics has been extensively utilised in medicine as a crucial approach for delineating field progress, identifying research objectives, and evaluating contributions. Despite a gradual increase in studies about the application of TCM in NAFLD, complete bibliometric assessments on this subject are still limited. In this study, bibliometric tools including R, VOSviewer, and CiteSpace were utilised to systematically analyze publications concerning the application of TCM in NAFLD between 2000 and 2024. The goal was to delineate the current research landscape and emerging trends, thereby providing valuable references to inform future investigations in this domain.

## 2 Materials and methods

### 2.1 Data collection

The data included in this analysis were primarily obtained from the Web of Science Core Collection (WoSCC), an authoritative bibliographic database indexing over 12,000 relevant scholarly publications. Compared to alternative databases such as Scopus, WoSCC provides comprehensive citation coverage and reliable data quality, making it a robust and widely used source for bibliometric analyses (23–25). The literature search in WoSCC was performed on April 12, 2025, using the following query: TS = (“traditional Chinese medicine” OR TCM OR “Chinese medicine” OR “Chinese herbal” OR “herbal medicine” OR “Chinese proprietary medicine*” OR decoction* OR powder* OR pill* OR “herbal formula*” OR “herbal extract*”) AND TS = (“nonalcoholic fatty liver disease” OR “non-alcoholic fatty liver disease” OR NAFLD OR “metabolic dysfunction-associated fatty liver disease” OR “metabolic associated fatty liver disease” OR MAFLD OR “nonalcoholic steatohepatitis” OR “non-alcoholic steatohepatitis” OR NASH OR “nonalcoholic hepatic steatosis” OR “non-alcoholic hepatic steatosis” OR NAFL). The search was confined to publications from January 1, 2000, to December 31, 2024, limited to articles and reviews published in English. A total of 855 records were acquired following the filtering process. The retrieved data, containing full records and their citations, were maintained in plain text files for ongoing analysis.

**Figure 1:**
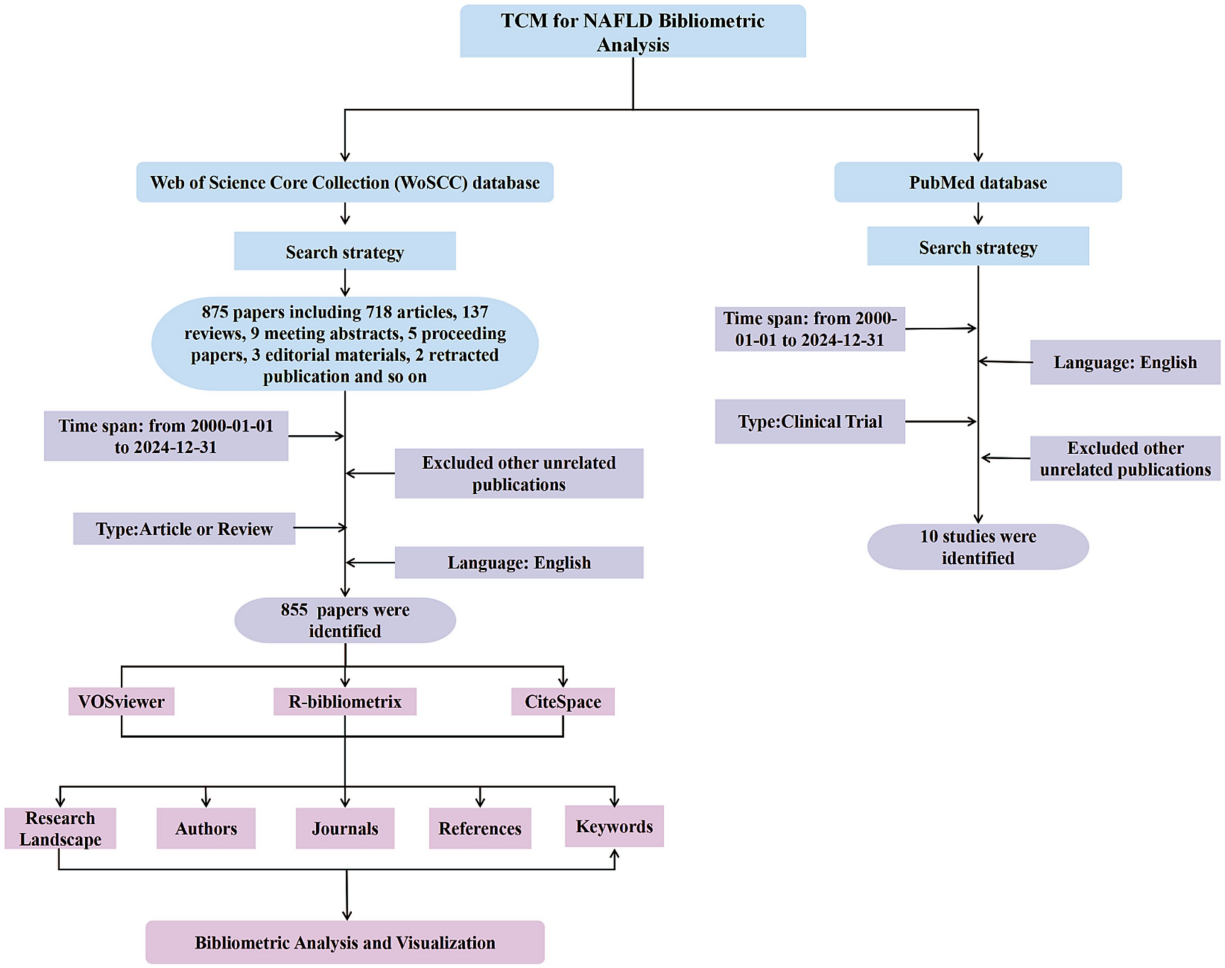
The process of literature search and selection.

In addition, to compensate for the limited inclusion of clinical studies in WoSCC, a supplementary search was conducted in the PubMed database using the same time frame and an equivalent search strategy. The PubMed query was: (“traditional Chinese medicine”[Title/Abstract] OR TCM[Title/Abstract] OR “Chinese medicine”[Title/Abstract] OR “Chinese herbal”[Title/Abstract] OR “herbal medicine”[Title/Abstract] OR “Chinese proprietary medicine”[Title/Abstract] OR decoction[Title/Abstract] OR powder[Title/Abstract] OR pill[Title/Abstract] OR “herbal formula”[Title/Abstract] OR “herbal extract”[Title/Abstract]) AND (“nonalcoholic fatty liver disease”[Title/Abstract] OR “non-alcoholic fatty liver disease”[Title/Abstract] OR NAFLD[Title/Abstract] OR “metabolic dysfunction-associated fatty liver disease”[Title/Abstract] OR “metabolic associated fatty liver disease”[Title/Abstract] OR MAFLD[Title/Abstract] OR “nonalcoholic steatohepatitis”[Title/Abstract] OR “non-alcoholic steatohepatitis”[Title/Abstract] OR NASH[Title/Abstract] OR “nonalcoholic hepatic steatosis”[Title/Abstract] OR “non-alcoholic hepatic steatosis”[Title/Abstract] OR NAFL[Title/Abstract]). A total of 10 eligible clinical studies were identified from this PubMed search for detailed analysis to enrich the clinical evidence base of this research. The overall retrieval and screening process is illustrated in Figure 1.

### 2.2 Data analysis

A variety of tools were applied for bibliometric analysis and visualization of the collected data. The R environment (version 4.4.1) was utilised with specific packages such as bibliometrix (version 4.3.0) for bibliometric computations and ggplot2 (version 3.5.2) for generating graphics. In addition, network mapping and knowledge structure visualization were performed using VOSviewer (version 1.6.20) and CiteSpace (version 6.2.4), respectively.

VOSviewer is a widely used tool for visualizing collaboration, co-citation, and keyword co-occurrence networks in scientific publications, capable of extracting and visualizing essential bibliometric information (19). This study employed VOSviewer to create visual network maps illustrating collaborations among countries and institutions, author collaborations, co-citations, and keyword co-occurrences. Bibliometrix is an R package developed for scientific knowledge mapping, including several bibliometric analysis capabilities, such as cooperation network development, research theme evolution tracking, and citation connection analysis (22). Bibliometrix was predominantly employed for the examination of topic evolution. CiteSpace, created by Chen, is a Java-based application extensively utilised in bibliometric and scientific knowledge mapping analysis (21). Using CiteSpace, we conducted journal dual-map overlay visualizations as well as analyses of citation bursts. The parameters for CiteSpace were configured as follows: Time frame: 2000–2024; duration of each segment = 1 year; selection criterion: g-index (*k* = 25). The ggplot2 program was utilised to visualise journal-related data, while Origin 2024 was employed to depict the disciplinary distribution within this study domain. Furthermore, Excel 2021 was employed to illustrate the distribution of principal financing agencies. The journal impact factors(IF) cited in this study were derived from the 2024 Journal Citation Reports.

## 3 Results

### 3.1 Overview of the bibliometric results

**Figure 2:**
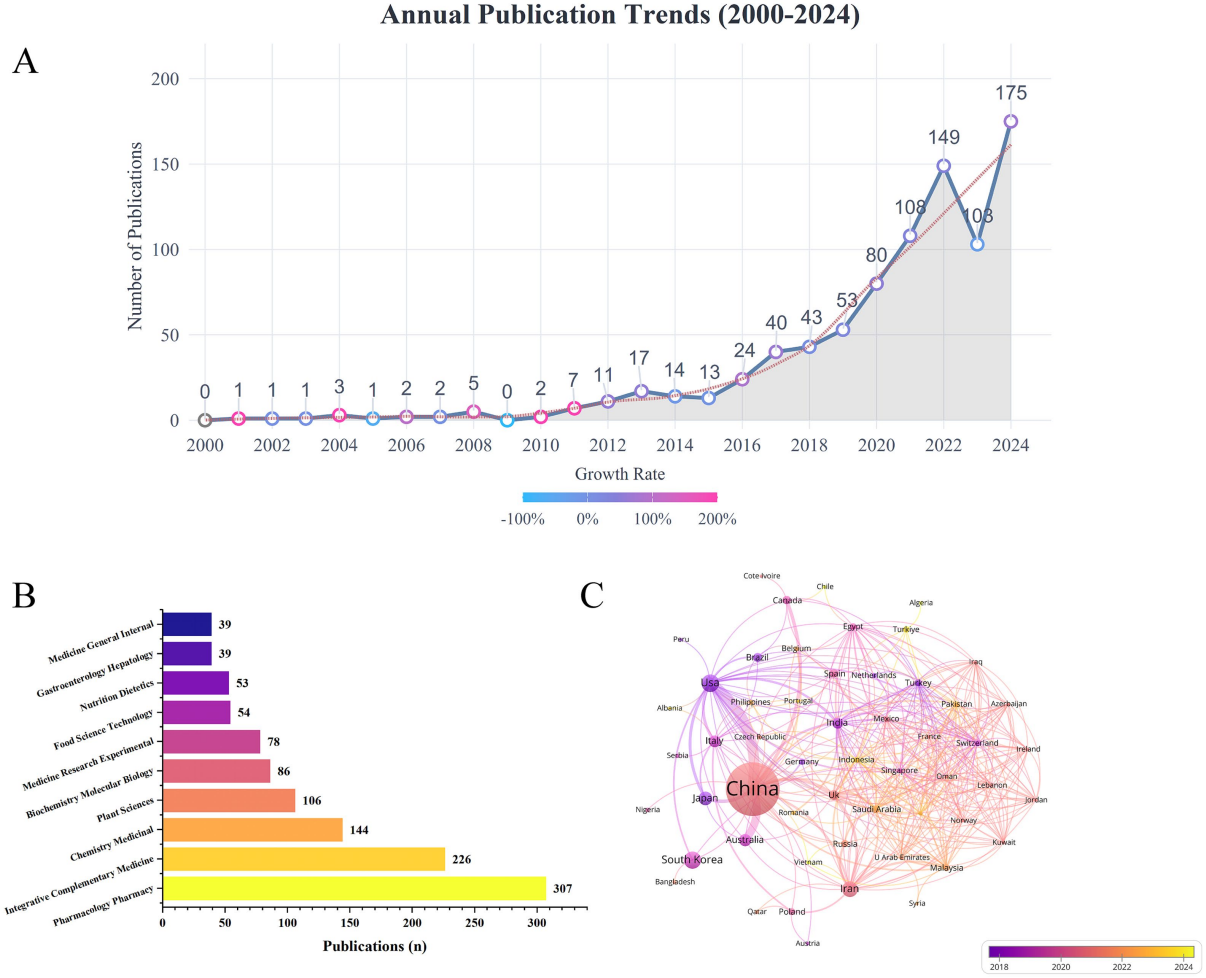
**(A)** Annual publication trends from 2000 to 2024. **(B)** Subject categories distribution. **(C)** Visualization map of country collaborations.

This study obtained material pertaining to TCM in NAFLD research from the WoSCC. Following rigorous screening, a total of 855 publications were incorporated, consisting of 718 original research pieces and 137 review papers. Figure 2A depicts the yearly publishing pattern from 2000 to 2024. From 2000 to 2016, the topic was in its nascent stage, characterized by a limited number of publications, culminating in a peak of 24 papers in 2016. Since 2017, research activity has significantly escalated, culminating in a peak of 175 articles in 2024. The red dashed line in the illustration denotes a fitted smoothing curve, illustrating the long-term trend of publication volume. The consistent rising trajectory signifies enduring advancement in TCM research within the NAFLD domain during the last 25 years. The hue of each circular data point indicates the annual growth rate in publication count: a more pinkish tint signifies a bigger increase. Years such as 2008, 2010, and 2011 exhibit clearly pink-toned dots, indicating significant growth relative to the preceding year. Figure 2B also displays the 10 principal subject groups of the included publications. The majority of papers on TCM in NAFLD studies were published in the domains of Pharmacology & Pharmacy (*n* = 307) and Integrative & Complementary Medicine (*n* = 226), followed by fields such as Medicinal Chemistry, Plant Sciences, and Biochemistry & Molecular Biology.

**Figure 3:**
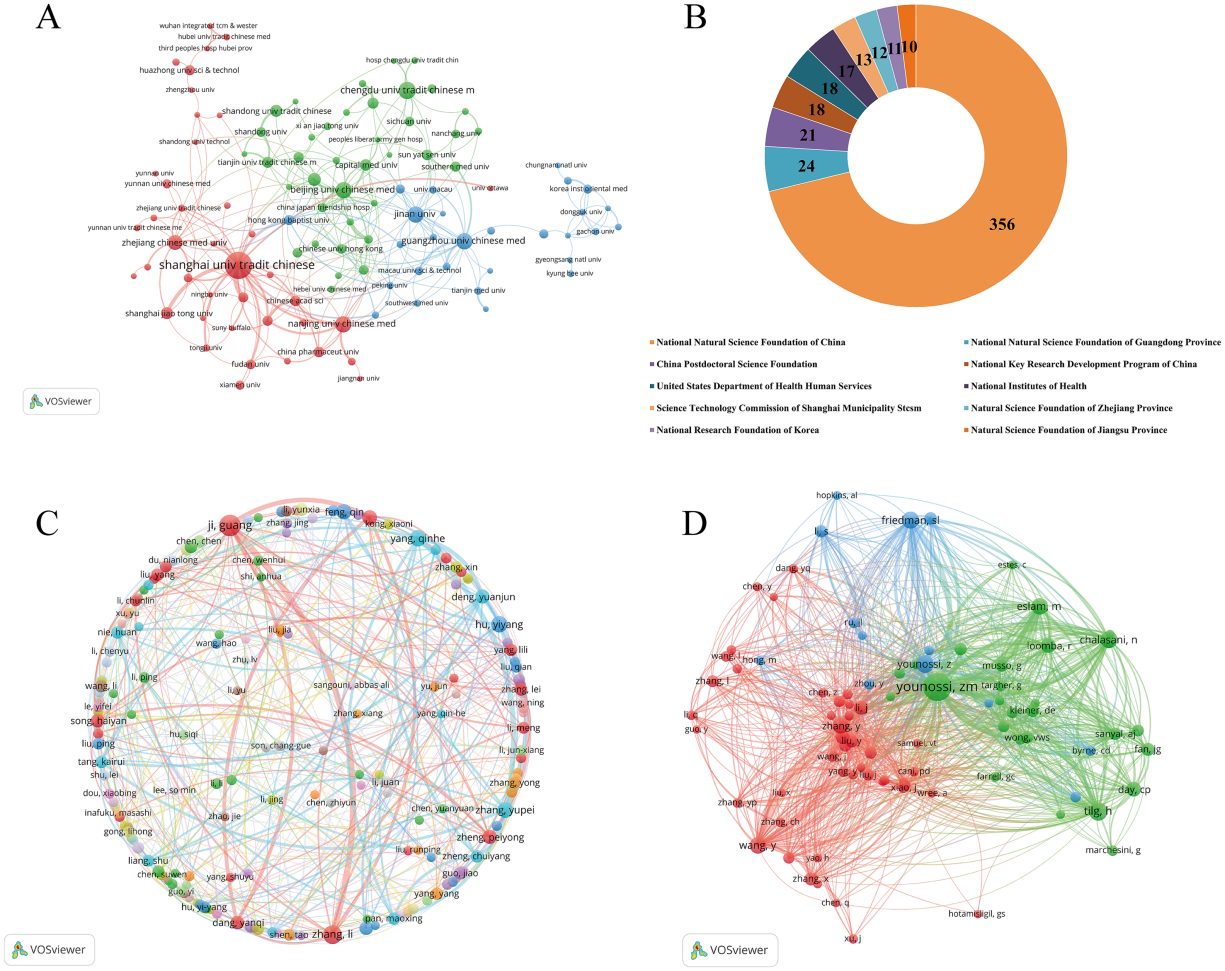
**(A)** Visualization map of institutional collaborations. **(B)** Distribution of funding agencies. **(C)** Visualization map of author collaborations. **(D)** Visualization map of co-cited authors.

A total of 1,062 research institutions from 57 countries engaged in investigations on TCM in NAFLD. Table 1 enumerates the 10 leading countries and institutions based on publication volume in this domain. China leads with 654 publications, representing 76.5% of the total output, followed by the United States (*n* = 49) and South Korea (*n* = 47). Figure 2C depicts the collaborative network among nations, highlighting active cooperation among various countries. The United States sustains robust cooperation relationships with China, Italy, Japan, South Korea, and Brazil. Moreover, China maintains strong alliances with Australia, Japan, Canada, and the United Kingdom. Shanghai University of Traditional Chinese Medicine has the highest number of publications (*n* = 103, 12.0%), followed by Chengdu University of Traditional Chinese Medicine (*n* = 37, 4.3%) and Beijing University of Chinese Medicine (*n* = 32, 3.7%). All 10 institutions with the highest publication counts in Table 1 are located in China, underscoring China’s preeminent position in this research domain. Figure 3A depicts active cooperation among institutions; for instance, Shanghai University of Traditional Chinese Medicine sustains strong cooperative ties with Nanjing University of Chinese Medicine, Zhejiang Chinese Medical University, and Shanghai Jiao Tong University. Figure 3B additionally displays the 10 principal funding bodies that endorse research on TCM in NAFLD. The National Natural Science Foundation of China leads with 356 sponsored projects, followed by the National Natural Science Foundation of Guangdong Province (*n* = 24) and the China Postdoctoral Science Foundation (*n* = 21). Of the 10 leading financing agencies, seven are situated in China, while the other three are from the United States and South Korea. The findings indicate that China’s substantial research output in this domain is intricately connected to its considerable funding, with significant financial resources being pivotal in facilitating research advancement.

**Table 1:** Top 10 most productive countries and affiliations in the field of TCM in NAFLD.

Rank	Country	Counts	Affiliation	Counts
1	China	654 (76.5%)	Shanghai University of Traditional Chinese Medicine(China)	103 (12.0%)
2	USA	49 (5.7%)	Chengdu University of Traditional Chinese Medicine(China)	37 (4.3%)
3	South Korea	47 (5.5%)	Beijing University of Chinese Medicine(China)	32 (3.7%)
4	Iran	38 (4.4%)	Guangzhou University of Chinese Medicine(China)	32 (3.7%)
5	Japan	27 (3.2%)	Jinan University(China)	31 (3.6%)
6	Australia	21 (2.5%)	Nanjing University of Chinese Medicine(China)	31 (3.6%)
7	Italy	19 (2.2%)	Zhejiang Chinese Medical University(China)	25 (2.9%)
8	India	17 (2.0%)	China Academy of Chinese Medical Sciences(China)	19 (2.2%)
9	UK	13 (1.5%)	Shanghai Jiao Tong University(China)	13 (0.8%)
10	Brazil	10 (1.2%)	Shandong University of Traditional Chinese Medicine(China)	12 (0.7%)

### 3.2 Authors and co-cited authors

A total of 5,072 researchers have participated in studies on TCM in NAFLD. Table 2 indicates that 10 writers have produced over eight publications, all linked with institutes in China. Ji G is the most prolific author with 29 publications, followed by Zhang L with 20 and Yang QH with 14. Figure 3C shows that some authors have established close collaborative relationships. For instance, Ji G regularly partnered with Zhang L, Dang YQ, Zhou WJ, and Song HY. Younossi ZM from the United States ranks first among co-cited authors with 287 citations, signifying his pivotal influence in this study domain. He is succeeded by Tilg H (*n* = 129) and Younossi Z (*n* = 119). In the co-citation network, researchers from the United States lead with half of the top 10 co-cited authors, followed by contributors from China, Austria, Australia, and the UK. Figure 3D illustrates robust cooperation among prominent authors, highlighting significant linkages between Younossi ZM and other notable academics, including Tilg H, Eslam M, and Chalasani N.

**Table 2:** Top 10 most published authors and co-cited authors in the field of TCM in NAFLD.

Rank	Author	Country	Counts	Co-cited author	Country	Citations
1	Ji G	China	29	Younossi ZM	USA	287
2	Zhang L	China	20	Tilg H	Austria	129
3	Yang QH	China	14	Younossi Z	USA	119
4	Hu Y	China	13	Chalasani NP	USA	115
5	Zhang Y	China	13	Friedman SL	USA	113
6	Feng Q	China	12	Wang Y	China	105
7	Zhou WJ	China	11	Eslam M	Australia	104
8	Deng YJ	China	10	Zhang Y	China	99
9	Zheng PY	China	10	Loomba R	USA	87
10	Li YX	China	9	Day CP	UK	84

### 3.3 Journals and cited journals

**Figure 4:**
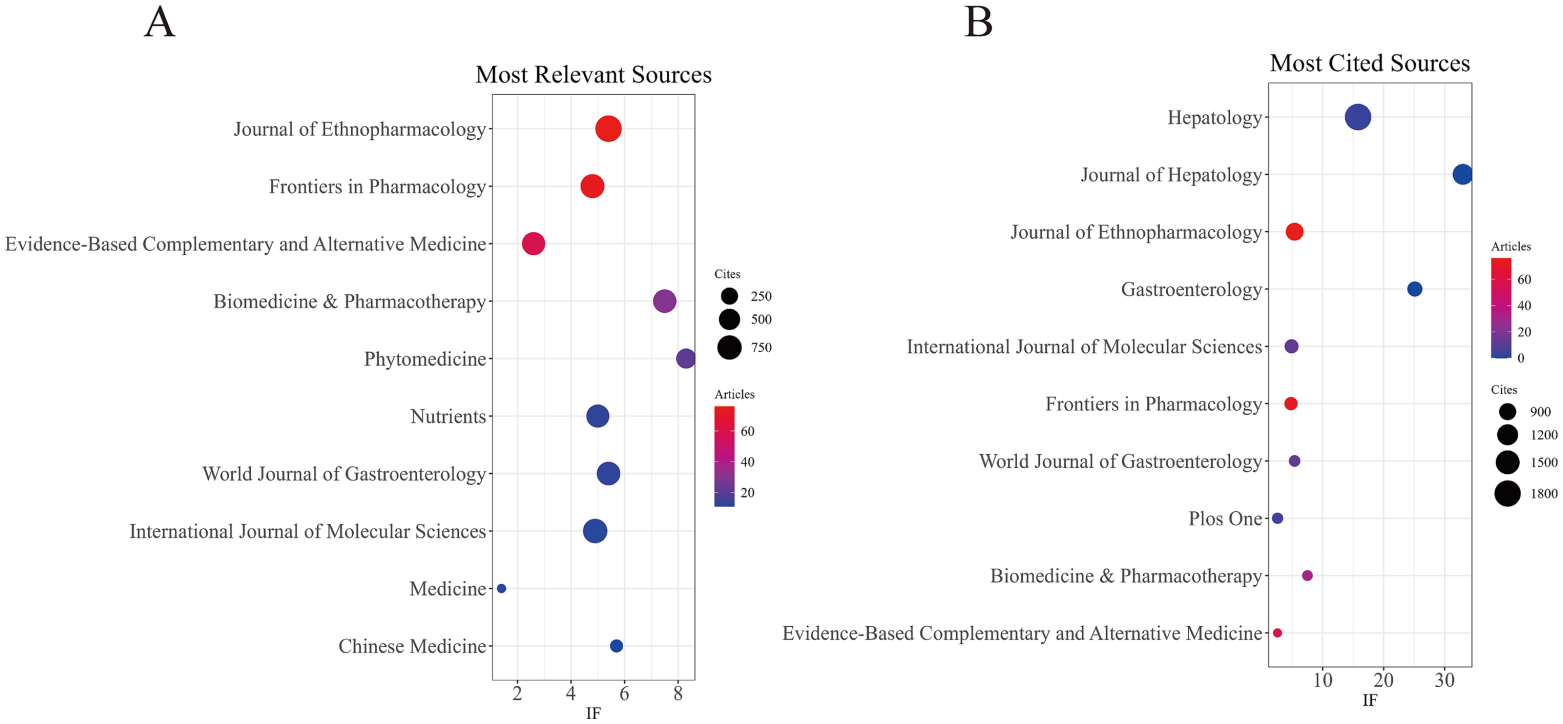
**(A)** Most relevant journals. **(B)** Most cited journals.

In the last 25 years, 281 scholarly journals have disseminated research on TCM in NAFLD. Table 3 and Figure 4A enumerate the 10 leading journals by publishing volume, each contributing over 10 articles. The Journal of Ethnopharmacology placed first with 76 publications, followed by Frontiers in Pharmacology with 74 papers and Evidence-Based Complementary and Alternative Medicine with 59 publications. Significantly, eight of these journals are designated as Q1 journals in the Journal Citation Reports, reflecting the superior quality and scholarly impact of research in this domain. Table 4 and Figure 4B indicate that the 10 most referenced journals have each garnered in excess of 600 citations. Hepatology has 1,811 citations and an IF of 15.8, followed by the Journal of Hepatology with 1,174 citations and an IF of 33.0, and the Journal of Ethnopharmacology with 950 citations and an IF of 5.4. These data indicate that while certain high-impact journals publish fewer articles in this domain, they garner considerable citations, demonstrating their great academic influence.

**Table 3:** Top 10 Most Productive Journals in the Field of TCM in NAFLD.

Rank	Journal	Counts	IF	Citations	Q
1	Journal of Ethnopharmacology	76	5.4	950	q1
2	Frontiers in Pharmacology	74	4.8	721	q1
3	Evidence-Based Complementary and Alternative Medicine	59	2.6	652	q3
4	Biomedicine & Pharmacotherapy	30	7.5	662	q1
5	Phytomedicine	21	8.3	409	q1
6	Nutrients	13	5.0	629	q1
7	World Journal of Gastroenterology	13	5.4	674	q1
8	International Journal of Molecular Sciences	12	4.9	746	q1
9	Medicine	12	1.4	61	q2
10	Chinese Medicine	11	5.7	106	q1

**Table 4:** Top 10 Most Cited Journals in the Field of TCM in NAFLD.

Rank	Journal	Citations	IF	Counts	Q
1	Hepatology	1811	15.8	3	q1
2	Journal of Hepatology	1,174	33.0	0	q1
3	Journal of Ethnopharmacology	950	5.4	76	q1
4	Gastroenterology	791	25.1	0	q1
5	International Journal of Molecular Sciences	746	4.9	12	q1
6	Frontiers in Pharmacology	721	4.8	74	q1
7	World Journal of Gastroenterology	674	5.4	13	q1
8	Plos One	669	2.6	5	q2
9	Biomedicine & Pharmacotherapy	662	7.5	30	q1
10	Evidence-Based Complementary and Alternative Medicine	652	2.6	59	q3

**Figure 5:**
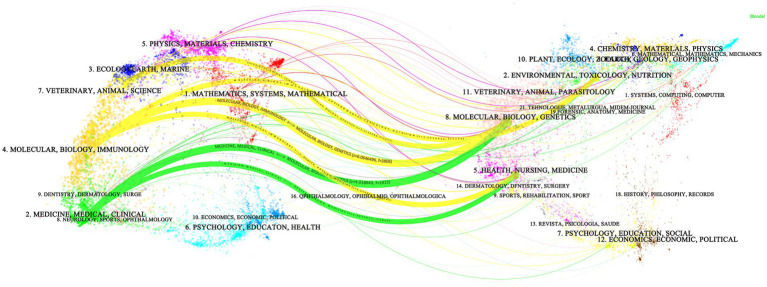
Dual-map overlay of journals.

Figure 5 illustrates the dual-map overlay of articles pertaining to research on TCM in NAFLD. The journals that are citing are presented on the left, while the journals that are cited are displayed on the right. Coloured citation paths show the primary channels of knowledge, with thicker paths signifying higher citation frequency. Research in Medicine, Medical and Clinical journals tends to cite literature from the Molecular, Biology and Genetics category, highlighting the close connection between clinical practice and molecular research foundations. Publications in Molecular, Biology and Immunology journals cite not only Molecular, Biology and Genetics but also Environmental, Toxicology and Nutrition and Health, Nursing and Medicine, revealing cross-disciplinary knowledge flows within the overlay map. A citation trajectory from Veterinary, Animal and Science to Molecular, Biology and Immunology is also evident, underscoring the linkage between animal-based research and molecular immunology in this domain. Overall, the dual-map overlay delineates a knowledge structure centered on molecular biology, connecting basic scientific research with clinical medicine while reflecting disciplinary integration across fields.

### 3.4 References with strong citation bursts

**Figure 6:**
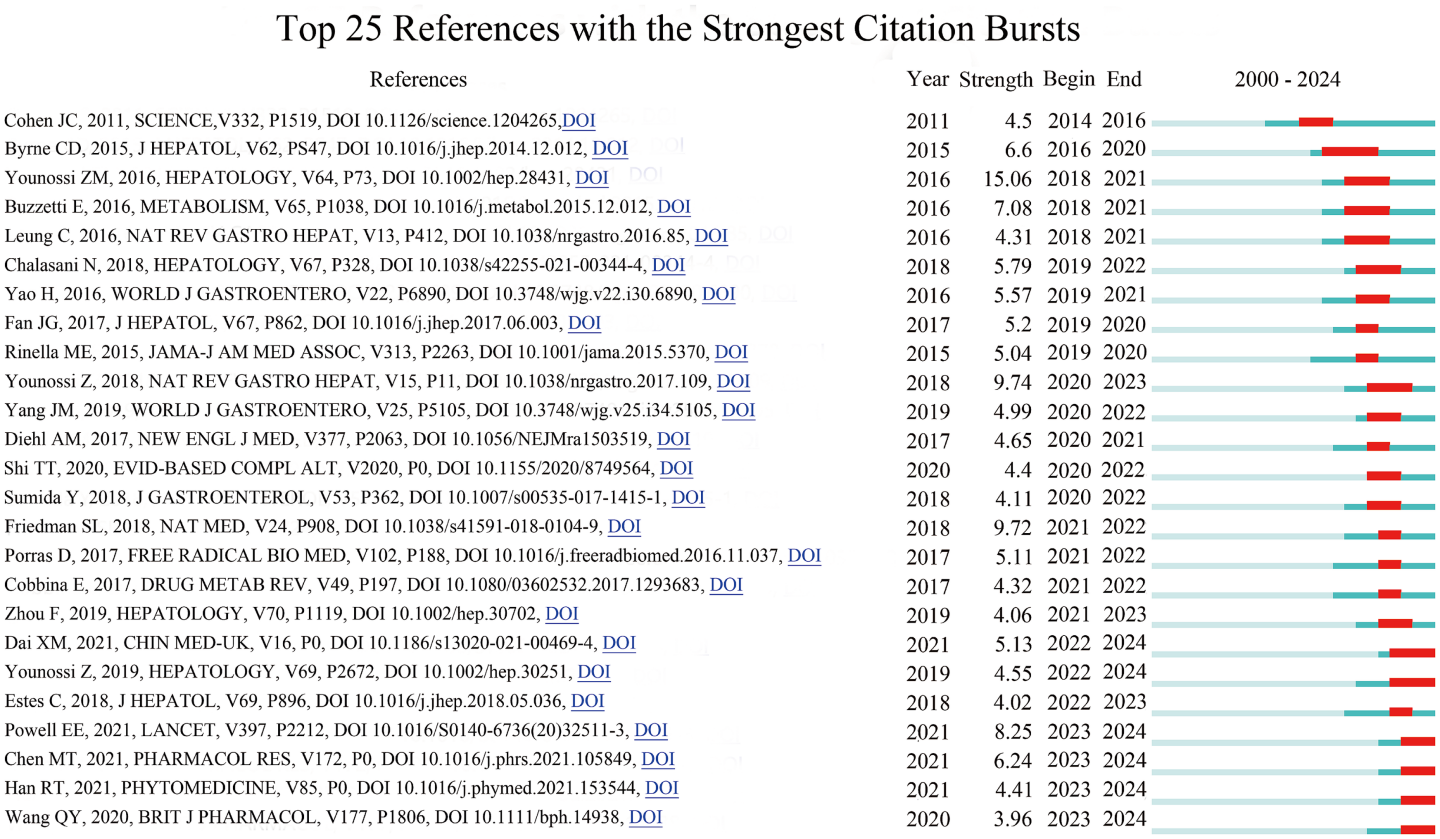
Top 25 references with the strongest citation bursts.

To pinpoint the most significant studies on TCM in NAFLD over time, we applied CiteSpace for citation burst analysis of relevant references. Between 2000 and 2024, 25 references exhibiting strong citation bursts were identified, with burst intensities varying from 3.96 to 15.06 (Figure 6; Supplementary Table S1). Figure 6 illustrates the temporal distribution of citation dynamics for these references. The blue segments represent the overall time span during which each reference was cited, whereas the red segments indicate periods during which citation frequency increased markedly, reflecting heightened academic attention within those specific intervals. The three references with the strongest citation bursts were “Global epidemiology of nonalcoholic fatty liver disease: Meta-analytic assessment of prevalence, incidence, and outcomes” (strength: 15.06), “Global burden of NAFLD and NASH: trends, predictions, risk factors and prevention” (strength: 9.74), and “Mechanisms of NAFLD development and therapeutic strategies” (strength: 9.72). These studies are all review articles, with citation-active periods primarily concentrated between 2018 and 2023. These reviews create the wider scientific framework that supports further research into TCM-based therapies for NAFLD by concentrating on the disease’s worldwide epidemiology, pathophysiology, and therapeutic approaches.

In addition, more recent citation bursts emerging during 2023–2024 provide insight into the current research priorities of the field. These include the following: “Non-alcoholic fatty liver disease,” “Traditional Chinese medicine in the treatment of nonalcoholic steatohepatitis,” “Naringenin attenuates non-alcoholic fatty liver disease by down-regulating the NLRP3/NF-κB pathway in mice,” and “Si Miao Formula attenuates non-alcoholic fatty liver disease by modulating hepatic lipid metabolism and gut microbiota.” When taken as a whole, these research show a growing focus on unraveling the underlying molecular and mechanistic processes involved in TCM’s therapeutic effects in NAFLD.

Among these recent burst references, two review articles provide comprehensive academic syntheses of the field. The review addressing the global status of NAFLD systematically outlines the complex pathological background and clinical challenges associated with the disease (26), while the review focusing on TCM in the treatment of NASH further highlights the therapeutic potential and mechanistic basis of TCM interventions (27). At the same time, two experimental animal studies have advanced mechanistic understanding at the molecular level. The classical formula Si Miao Formula demonstrated multi-target regulatory effects in metabolic dysfunction. Experimental findings indicated that this formula reduced lipid accumulation by downregulating genes involved in hepatic fatty acid biosynthesis and simultaneously increased the relative abundance of beneficial gut microbiota, including *Akkermansia muciniphila* (28). These findings bolster the idea that TCM may play a part in regulating the gut-liver axis and imply that one of the key mechanisms by which TCM treats NAFLD is the control of gut microbiota. Similarly, by downregulating the NLRP3/NF-κB signalling pathway, the monomeric molecule naringenin was found to drastically reduce hepatic inflammation and lipid deposition (29). This work further provides molecular-level evidence supporting the important role of inflammatory signalling pathways in TCM-mediated intervention in NAFLD.

Overall, the 25 burst references can be broadly classified into three major categories: (1) Epidemiological and disease pattern studies, primarily focusing on the prevalence, disease burden, and public health implications of NAFLD; (2) Mechanistic and therapeutic strategy research, addressing the “multiple-hit” hypothesis, gut microbiota dysbiosis, lipid metabolic dysfunction, inflammation-related signalling pathways, and potential therapeutic approaches; (3)TCM intervention and mechanistic studies, including examinations of monomers and traditional formulations like quercetin, Si Miao Formula, and naringenin, as well as their regulatory mechanisms in NAFLD.

### 3.5 Keyword clusters and evolution

Keyword clustering analysis facilitates the discovery of research hotspots and evolutionary trends, providing a comprehensive understanding of the present status and future directions in the field. This study conducted a keyword visualization analysis via VOSviewer, resulting in the identification of 1870 keywords. Table 5 enumerates the top 20 keywords with the highest frequency of occurrence, including significant terms such as gut microbiota, network pharmacology, inflammation, insulin resistance, lipid metabolism, and obesity (excluding retrieval terms)

**Table 5:** Top 20 keywords in the field of TCM in NAFLD.

Rank	Keyword	Occurrences
1	nonalcoholic fatty liver disease	477
2	traditional chinese medicine	152
3	gut microbiota	79
4	network pharmacology	71
5	inflammation	67
6	insulin resistance	49
7	lipid metabolism	48
8	obesity	48
9	oxidative stress	45
10	high-fat diet	25
11	hepatic steatosis	23
12	mechanism	23
13	ampk	21
14	molecular docking	20
15	autophagy	19
16	metabolic syndrome	19
17	meta-analysis	18
18	metabolomics	18
19	diabetes	17
20	type 2 diabetes	17

**Figure 7:**
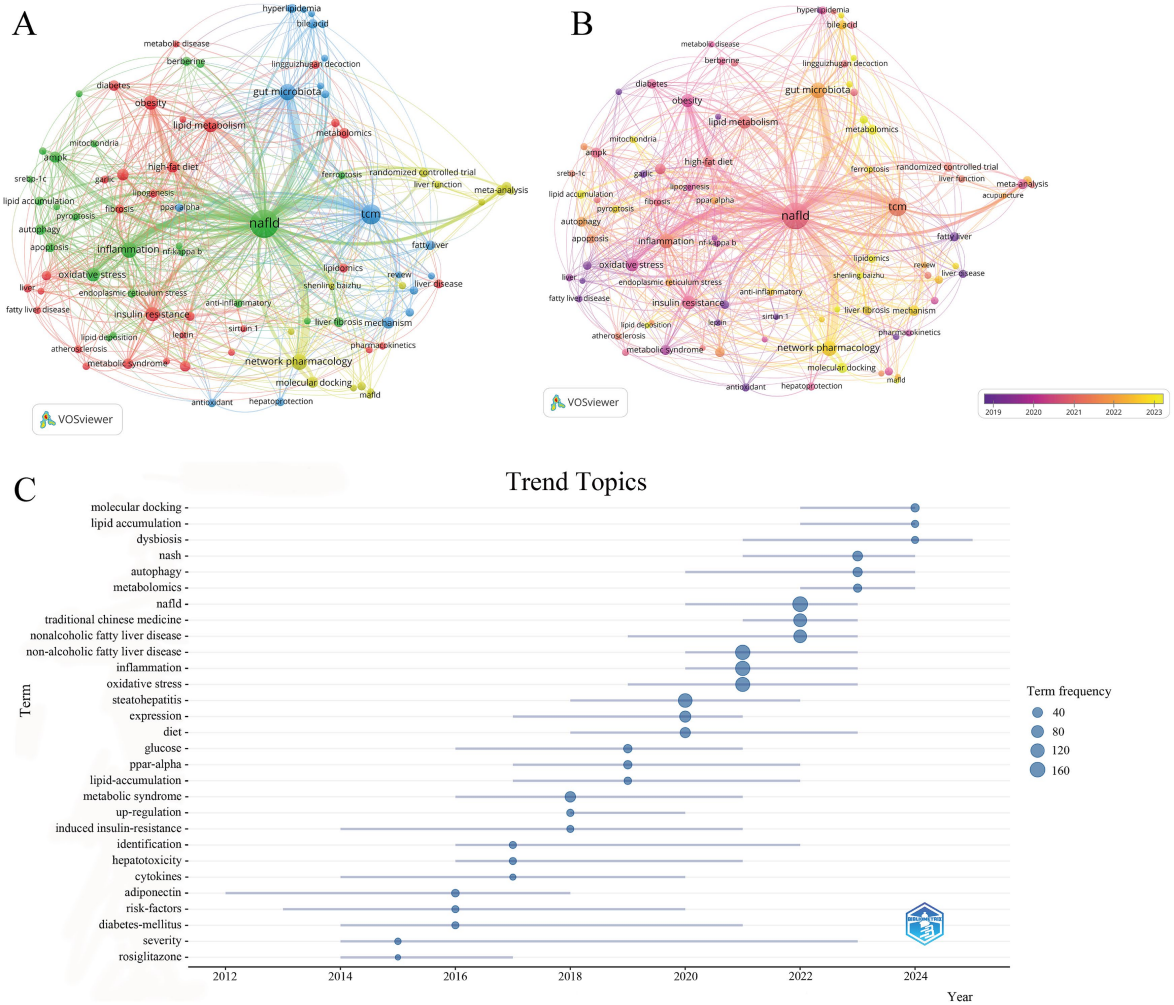
**(A)** Visualization map of keyword co-occurrence. **(B)** Overlay visualization map of keyword co-occurrence. **(C)** Trending topics.

Figures 7A,B depicts the relationships among terms, representing VOSviewer’s network visualization and overlay visualization, respectively. The thickness of the linkages denotes the frequency of co-occurrence between keywords, with thicker lines signifying greater correlations. The keywords are grouped into four clusters in Figure 7A, each of which is identified by a distinct color. The yellow cluster primarily pertains to TCM treatment of NAFLD and related research methodologies, consisting of 15 terms. Key terms include: “network pharmacology,” “molecular docking,” “randomized controlled trial,” “acupuncture,” “chaihu shugan powder,” “shenling baizhu powder,” and “meta-analysis.” The blue cluster focuses on the modulation of gut microbiota by TCM in NAFLD, consisting of 20 terms. Key terms include: “gut microbiota,” “mechanism,” “treatment,” “bile acid,” “short-chain fatty acids,” and “gut-liver axis.” The red cluster primarily focuses on the regulatory effects of TCM on lipid metabolism abnormalities associated with NAFLD, consisting of 31 terms. Key terms include: “lipid metabolism,” “obesity,” “high-fat diet,” “insulin resistance,” “PPAR gamma,” “lipogenesis,” and “lingguizhugan decoction.” The green cluster mainly centers on the modulation of inflammatory responses and related signalling pathways by TCM in the context of NAFLD, consisting of 24 terms. Key terms include: “inflammation,” “anti-inflammatory,” “oxidative stress,” “NF-kappa B,” “berberine,” “AMPK,” and “ferroptosis.”

We investigated the temporal evolution of keywords using overlay visualization in order to gain a deeper comprehension of the evolution of this field of study. The most common terms surrounding 2019 include “obesity,” “insulin resistance,” “oxidative stress,” “fatty liver,” and “meta-analysis,” as seen in Figure 7B. In contrast, research conducted around 2023 has focused on terms like “gut microbiota,” “bile acid,” “metabolomics,” “network pharmacology,” “molecular docking,” and “mechanism,” indicating a growing interest in the gut microbiota and the mechanisms that underlie it. The investigation of TCM remedies for NAFLD has been made possible by the use of network pharmacology and molecular docking.

To show the development of study subjects throughout different time periods, we created a theme evolution map (Figure 7C) using the Bibliometrix package in R. The research can be broadly divided into two sections. In the initial phase (2012–2017), the quantity of papers was rather modest, with primary keywords including “rosiglitazone,” “diabetes-mellitus,” “adiponectin,” and “risk-factors,” indicating an emphasis on metabolic diseases and associated pharmacological treatments for NAFLD. Since 2018, terms such as “oxidative stress,” “inflammation,” “ppar-alpha,” and “lipid accumulation” have become increasingly prevalent, indicating a transition in study focus towards a more comprehensive investigation of fundamental mechanisms. The current research focal points in this domain are mainly concentrated on “molecular docking,” “lipid accumulation,” and “dysbiosis,” signifying that the regulation of lipid metabolism, gut microbial balance, and the application of related methodological approaches have become important research directions in TCM treatment of NAFLD.

Taken together, the results of the keyword clustering and thematic evolution analyses indicate that current research on TCM concerning NAFLD primarily emphasises the following aspects: (1) the protective effects and therapeutic potential of TCM against NAFLD; (2) the utilisation of network pharmacology, molecular docking, and other analytical methodologies to elucidate the underlying mechanisms of TCM; (3) a comprehensive investigation of TCM’s therapeutic mechanisms, particularly regarding gut microbiota, lipid metabolism, and inflammatory pathways.

### 3.6 Clinical trial analysis

To evaluate the clinical translation landscape, a supplementary search was conducted in the PubMed database, ultimately including 10 eligible TCM clinical trials. Compared to the total volume of 855 publications from WoSCC, the number of TCM clinical trials appears relatively scarce. Despite the limited number of clinical trials, we found that the research focus of these trials demonstrated consistency with the core hotspots identified in the WoSCC analysis. Specifically, Berberine (BBR), a core monomer hotspot in the WoSCC analysis, was also highly represented in the 10 clinical trials, appearing 3 times. Similarly, Lingguizhugan Decoction (LGZG), as a representative formula hotspot in WoSCC, was also validated in these 10 trials. Detailed information on these clinical trials is provided in Supplementary Table S2.

The sample size of the individual studies ranged from 50 to 300 participants. These clinical trials consistently demonstrated improvements in metabolic-related indices and liver function across various control settings, indicating the potential therapeutic benefit of TCM in NAFLD patients. Additionally, clinical research has shown that several herbal formulae have been linked to changes in the composition of the gut microbiota, with improvements in AST and ALT levels being correlated with changes in the microbiota (30). In addition, these studies observed reductions in inflammatory markers, including TNF-*α* and IL-8, as well as improvements in lipid profiles and metabolic parameters such as HOMA-IR (31, 32). These clinical findings suggest that TCM interventions may contribute to disease amelioration in NAFLD patients through gut microbiota modulation, attenuation of inflammatory responses, and improvement of lipid metabolism. Importantly, these TCM therapies demonstrated favourable safety profiles, with reported adverse events being predominantly mild and transient gastrointestinal discomfort. Overall, these clinical results complement the existing evidence derived primarily from animal studies and provide preliminary human-based support for the potential involvement of gut microbiota regulation, inflammatory modulation, and lipid metabolic improvement in the therapeutic effects of TCM against NAFLD.

## 4 Discussion

### 4.1 General information

We performed a bibliometric study of 855 pertinent papers from 2000 to 2024 to elucidate the current status and emerging trends in the use of TCM for nonalcoholic NAFLD. The data demonstrate that, despite minor fluctuations in the annual publication count, the overarching trend has been a consistent increase, signifying heightened academic interest in this domain. This development underscores the favourable study opportunities and potential of TCM in addressing NAFLD, indicating that forthcoming investigations in this domain are expected to proliferate.

China was the foremost contributor, publishing 654 papers, followed by the United States and South Korea. Significantly, all of the leading 10 most prolific institutions and authors are situated in China. Ji G is the most prolific author, while Shanghai University of Traditional Chinese Medicine is the most influential research institution, both significantly contributing to the advancement of this discipline. These findings highlight China’s preeminence in TCM-related NAFLD research, demonstrating a robust foundation in both fundamental and clinical TCM investigations, as well as the probable influence of favourable government policies and significant research funding (33). Conversely, the United States displayed a more elevated degree of international collaboration, while China revealed a more restricted worldwide cooperation network. This observation indicates a necessity to improve China’s foreign partnerships to expedite the global advancement of TCM concerning NAFLD. The most often cited author was Zobair M. Younossi from the United States, whose contributions to NAFLD epidemiology, diagnostic criteria, and worldwide disease burden evaluations have established a crucial theoretical framework for TCM-related research in this field. Research on TCM treatment for NAFLD has been published in 281 journals. Prominent journals in this field include Journal of Ethnopharmacology, Frontiers in Pharmacology, Evidence-Based Complementary and Alternative Medicine, and Biomedicine & Pharmacotherapy. Hepatology and Journal of Hepatology were the most often cited journals, signifying the significant effect and importance of their published publications to the advancement of TCM research in NAFLD.

### 4.2 Hot spots and development trends

Through an extensive examination of citation bursts, keyword clustering, and thematic progression, we discerned the principal research trajectories and nascent trends in TCM concerning NAFLD. Contemporary study in this field predominantly focuses on the following principal areas:

#### 4.2.1 Mechanistic insights into the effects of TCM on NAFLD

Our data indicate that TCM has emerged as a prominent research focus in this domain by modulating NAFLD through the regulation of intestinal flora, lipid metabolism, and inflammatory responses. Disruption in gut microbiota can facilitate the onset and advancement of NAFLD via multiple mechanisms, such as increasing intestinal permeability, impairing bile acid metabolism, modifying short-chain fatty acids (SCFAs) production, and triggering inflammatory responses (34). TCM may exert anti-NAFLD effects by modulating the diversity and abundance of gut microbiota and restoring intestinal microecological balance. For example, compound herbal formulations such as Shenling Baizhu San, Quzhi Ruangan Fang, and Gexia Zhuyu Tang have demonstrated the ability to regulate gut microbiota and ameliorate metabolic disorders in animal models (35). Moreover, advances in metabolomics have underscored the critical roles of gut microbial metabolites, including bile acids and SCFAs, in the pathogenesis of NAFLD (36). Specifically, Zhuyu Pill and Pien Tze Huang have been reported to alleviate NAFLD by modulating bile acid metabolism (37, 38), while Zuogui-Jiangtang-Qinggan-Fang improves lipid metabolism disorders through adjustment of SCFAs composition (39). Notably, the compound formula LGZG not only reshapes the intestinal microbiota but also concurrently regulates SCFAs and bile acid metabolism, thereby exerting multi-faceted intervention effects on NAFLD (40). These findings suggest that targeting gut microbiota modulation represents a promising therapeutic strategy for NAFLD treatment in TCM.

Dysregulated lipid metabolism is a fundamental pathogenic mechanism of NAFLD and a primary focus of TCM treatments. The AMP-activated protein kinase (AMPK)-mediated metabolic signalling system has become a prominent focus of research among several regulatory pathways. AMPK is an essential kinase that plays a vital role in regulating energy homeostasis. Upon phosphorylation activation, it can enhance lipid oxidation and suppress lipid synthesis, thus controlling hepatic lipid metabolism and mitigating hepatic fat formation. Furthermore, AMPK activation alleviates oxidative stress and inflammatory reactions by diminishing reactive oxygen species and pro-inflammatory cytokines (41). A substantial body of data has shown that TCM can have therapeutic effects on NAFLD via activating the AMPK signalling system. The Tiaogan Jiejiu Tongluo formula, salvianolic acid A (the principal active compound of *Salvia miltiorrhiza*), and the alkaloid neferine (derived from *Nelumbo nucifera*) have been documented to activate AMPK, modulate lipid metabolism, diminish hepatic fat accumulation, and suppress inflammatory responses, thus significantly enhancing the progression of NAFLD (42–44).

Simultaneously, the inflammatory response, a pivotal pathogenic factor in the transition from NAFLD to NASH, is another significant therapeutic target of TCM. TCM can inhibit hepatic inflammation via many mechanisms, chiefly by regulating classical inflammatory signalling pathways like NF-κB. Various herbal constituents and formulations, such as celastrol, lignans derived from Schisandra chinensis, and the JianPi-QingHua combination, have demonstrated considerable anti-inflammatory properties. These drugs can impede the activation of the NF-κB pathway, reduce the expression of pro-inflammatory cytokines, and thereby mitigate hepatic inflammation (45–47).

It is crucial to acknowledge that the genesis and progression of NAFLD are not driven by a singular etiological component, but rather arise from a complex interplay of interconnected pathogenic pathways. These systems are not independent; rather, they are intricately interwoven and mutually regulated. For instance, dysbiosis of gut microbiota may affect the synthesis of short-chain fatty acids and bile acids, thus impairing lipid metabolism and triggering inflammatory responses (48, 49). Lipid metabolic problems, especially increased saturated fatty acid levels, can subsequently induce chronic inflammation by activating signalling pathways such as TLR4, the NLRP3 inflammasome, and oxidative stress. The resultant inflammatory condition may additionally hinder hepatic lipid metabolism (50) and disrupt gut microbial equilibrium (51). TCM often demonstrates therapeutic effects on NAFLD by regulating many targets and pathways, concurrently influencing various fundamental pathological processes. This comprehensive method of action corresponds effectively with the complex and interconnected character of NAFLD pathophysiology, providing benefits that surpass those of “single-mechanism” therapy. Current research predominantly examines the effects of TCM on specific targets or pathways, thus constraining our comprehension of its inherent multi-target intervention characteristic and inadequately representing its extensive regulatory impact on the intricate pathological network of NAFLD. Consequently, future investigations should prioritise the multi-target intervention mechanisms of TCM to clarify its comprehensive therapeutic mode of action in NAFLD and to further our comprehension of its fundamental pharmacological mechanisms.

#### 4.2.2 Identification of potential TCM candidates for NAFLD treatment

In the keyword analysis, Berberine and Lingguizhugan Decoction emerged as the most often cited phrases connected to TCM, denoting a bioactive component and a classical herbal formulation, respectively. This underscores their significant research focus and possible therapeutic efficacy in addressing NAFLD, an observation substantiated by our clinical trial analysis.

Berberine (BBR), an isoquinoline alkaloid, is a prominent bioactive constituent of Rhizoma coptidis, a traditional Chinese medicinal herb. It demonstrates a broad spectrum of pharmacological actions, encompassing anti-inflammatory, antioxidant, lipid-lowering, hypoglycemic, and gut microbiota-regulating activities (52, 53). In recent years, BBR has garnered heightened attention for its potential to alleviate NAFLD and its associated consequences. BBR promotes the SIRT1–FoxO1 signalling pathway, hence diminishing intracellular fat accumulation and total cholesterol levels. Furthermore, it targets the aldo-keto reductase AKR1B10 and affects the PPAR signalling pathway, consequently enhancing lipid metabolism disorders and insulin resistance (54). Furthermore, BBR exhibits substantial anti-inflammatory and antifibrotic properties. It mitigates hepatic inflammation by suppressing neutrophil activation, diminishing immune cell infiltration, and downregulating pro-inflammatory gene expression. It also regulates the activation of hepatic stellate cells and genes linked to cholangiocyte proliferation, hence inhibiting the advancement of liver fibrosis (55). BBR significantly influences the gut microecosystem. It modifies the gut microbiota composition by enhancing the prevalence of Bifidobacteria, Bacteroidetes, and Firmicutes, thereby promoting microbial equilibrium. This regulation enhances the production of tight junction proteins, fortifies intestinal barrier integrity, and alleviates hepatic inflammation and oxidative stress (56). BBR inhibits NAFLD progression via many pathways and targets, exhibiting a positive safety profile (57) and underscoring its strong therapeutic potential.

Evidence from randomized controlled trials consistently indicates that BBR significantly reduces hepatic fat content, improves liver enzymes, and ameliorates associated metabolic disorders in NAFLD patients (32, 58, 59). However, these studies also commonly highlight BBR’s primary limitations: namely, low bioavailability and mild gastrointestinal adverse events. Therefore, the future outlook for BBR should focus on exploring new drug delivery techniques, such as nanoparticle-based systems or salt-formulations, to address its pharmacokinetic obstacles and improve its clinical application value (60, 61).

Lingguizhugan Decoction (LGZG) is a historical traditional Chinese formulation consisting of Poria cocos, Cinnamomi Ramulus, Atractylodis Macrocephalae Rhizoma, and Glycyrrhizae Radix, initially recorded in the Golden Chamber Synopsis (62). It is conventionally employed to fortify the spleen, alleviate retained fluid, invigorate yang, and eradicate dampness. LGZG, a commonly employed herbal remedy, has been utilised for an extended period in the management of metabolic problems. Prior research indicates that LGZG markedly enhances liver function, diminishes serum lipid concentrations, mitigates oxidative stress, decreases insulin resistance, and inhibits inflammatory responses (63, 64). The advancing research highlights the potential of LGZG in regulating gut microbiota and its metabolites, which may be a crucial mechanism for its therapeutic effect on NAFLD. LGZG has been shown to enhance the prevalence of bacterial taxa associated with bile acid metabolism, including Bacteroides and Akkermansia, thus modulating bile acid composition and stimulating FXR/TGR5 signalling along the gut-liver axis. This facilitates bile acid balance while concurrently activating PPAR signalling to diminish hepatic lipid buildup and inflammation, hence enhancing liver function and intestinal barrier integrity (40, 62). Significantly, LGZG’s capacity to modify the gut microbiota can be conveyed through faecal microbiota transplantation, substantially mitigating obesity and hepatic steatosis generated by a high-fat diet in animal models (65).

A key randomized controlled trial included in our PubMed analysis further validated the beneficial efficacy of LGZG in the therapy of NAFLD (66). While recent research has identified various methods by which LGZG may aid in NAFLD, the precise bioactive constituents accountable for its therapeutic effects are still ambiguous. Furthermore, the synergistic interactions among its constituent herbs necessitate additional clarification. Subsequent research should concentrate on pinpointing the primary active ingredients and elucidating their functions and interactions within the formulation, thus establishing a basis for mechanistic understanding and precise use of multi-component TCM medicines.

It is significant that despite the variations in compositions of the previously listed herbal monomers and chemical prescriptions, they all have multi-target regulatory effects on NAFLD through various pathways, including the modulation of gut microbiota, lipid metabolism, and inflammatory responses. This corresponds effectively with the intricate pathophysiology features of NAFLD. Consequently, future selection of promising TCM-based therapy candidates should prioritise molecules that exhibit the capacity to modulate various pathways and targets, facilitating a more holistic and integrated approach to the multifactorial course of NAFLD. Furthermore, although numerous TCMs have demonstrated significant therapeutic potential in preclinical research, our clinical trial analysis reveals that the number of drugs that have truly entered clinical trials remains exceptionally scarce. Therefore, for those most promising candidates that have already shown clear efficacy in basic research, the progression to large-scale, multicenter randomized controlled trials must be accelerated to truly validate their clinical value.

#### 4.2.3 Network-based approaches in TCM research on NAFLD

Recent advancements in systems biology, bioinformatics, and computational biology have led to the emergence of network pharmacology (NP) as a crucial method for elucidating the multi-component, multi-target, and multi-pathway mechanisms of TCM (67). NP underscores a comprehensive approach to analyze the intricate relationships among diseases, genes, targets, and medications, which strongly corresponds with the TCM concept of multi-component synergy and multi-target intervention. Numerous studies have utilised NP techniques to investigate the underlying processes of TCM therapies for NAFLD. NP analysis systematically forecasted the therapeutic mechanisms of Chaihu Lizhong Decoction in combating NAFLD by identifying various active compounds and potential targets; protein–protein interaction network analysis underscored critical nodes such as AKT1 and IL6, while enrichment analyses indicated involvement predominantly via AGE-RAGE and IL-17 signalling pathways (68). Likewise, NP investigations on naringenin have shown its regulation of several key NAFLD targets, including AKT1, PPARG, and MAPK1, indicating pathways associated with lipid metabolism, viral infection, and AGE-RAGE signalling (69).

The combined use of NP with molecular docking, as well as *in vivo* and *in vitro* research, has become a prominent approach for clarifying the mechanisms of TCM formulae. Molecular docking, a recognised computer-aided structural analysis method extensively utilised in drug discovery and mechanistic investigations (70). facilitates the computational simulation of binding affinity and conformation between active TCM compounds and target proteins, thereby offering preliminary validation of NP predictions. Moreover, experimental validation of the regulatory impacts of active substances on essential target proteins and their participation in pertinent signalling networks bolsters the biological validity of these results. For instance, research on *Polygonum perfoliatum* L. utilised NP to ascertain its principal targets concentrated in the PI3K/AKT pathway. Subsequent UPLC/QE-HFX research and molecular docking elucidated major active components, including quercetin and baicalein, affirming their stable binding to essential targets such as AKT1 and PIK3R1. Animal studies indicated that PPL confers hepatoprotective benefits through the activation of the PI3K/AKT pathway and the mitigation of NAFLD phenotypes (71). With the rapid advancement of artificial intelligence (AI) and machine learning technologies, NP face new opportunities for development. AI techniques can enhance the efficiency and accuracy of active compound screening and target prediction, further facilitating the elucidation of complex disease mechanisms (67, 72). Consequently, future endeavours must concentrate on refining algorithms and incorporating multi-omics data to enhance the utilisation of NP in innovative TCM drug discovery and the thorough examination of its multi-target and multi-pathway processes.

### 4.3 Strengths and limitations

This study enhances comprehension of the existing research status and developmental tendencies of TCM concerning NAFLD, offering significant references for future enquiries. Nonetheless, many constraints warrant acknowledgement. The literature retrieval was confined to the Web of Science and PubMed databases, omitting additional databases, potentially leading to the exclusion of pertinent studies and thereby impacting the comprehensiveness of the material. Nonetheless, as a prevalent and exemplary high-quality database, Web of Science offers authoritative and reliable data, rendering our chosen dataset trustworthy. Secondly, only articles in the English language were considered, potentially neglecting significant research disseminated in other languages. The selection and logical arrangement of search terms may have resulted in the omission of relevant literature, exacerbated by the inconsistent application of related terminology across research, potentially impacting the thoroughness and precision of the retrieval. Finally, as a bibliometric method, this approach may inherently involve certain biases and risks of misinterpretation during data processing and result analysis. Notwithstanding these constraints, our analysis delivers a thorough overview of the present state, research focal points, and developmental trajectories in this domain, furnishing significant insights and direction for forthcoming research endeavours.

## 5 Conclusion

This study employed bibliometric analysis to systematically elucidate the research state, key subjects, and rising horizons for TCM in NAFLD. The results reveal that the principal findings and prospective trends in this domain predominantly encompass the following aspects:

- From 2000 to 2024, the total number of articles demonstrated an upward trajectory. China led in publication volume, dominating the research sector, followed by the United States and South Korea.
- Ji G is the most prolific author in this domain, whereas Younossi ZM holds the top position in co-citation frequency. The journals with the most significant publication volumes are Journal of Ethnopharmacology and Frontiers in Pharmacology, indicating their substantial engagement in TCM research. Prominent journals such as Hepatology and Journal of Hepatology signify the increasing global academic impact of this field.
- Mechanistic investigations of TCM in relation to NAFLD primarily concentrate on critical pathways, including gut microbiota regulation, lipid metabolism modulation, and inflammation attenuation. These mechanisms have emerged as significant research focal points and primary trajectories for future advancements in TCM therapy of NAFLD.
- Research on TCM therapies for NAFLD is expanding, with prominent drugs including BBR and LGZG garnering significant interest.
- Analytical methodologies such as network pharmacology are progressively utilised in NAFLD-related TCM research, emerging as essential technical approaches and focal points for clarifying the intricate mechanisms of TCM.

## Data Availability

Publicly available datasets were analyzed in this study. They were obtained from the Web of Science database, which can be accessed at: https://www.webofscience.com. Data were also obtained from the PubMed database, which can be accessed at: https://pubmed.ncbi.nlm.nih.gov/.

## References

[1] Golabi P Owrangi S Younossi ZM. Global perspective on nonalcoholic fatty liver disease and nonalcoholic steatohepatitis - prevalence, clinical impact, economic implications and management strategies. Aliment Pharmacol Ther. (2024) 59:S1–s9. doi: 10.1111/apt.17833, 38813821

[2] Wei S Wang L Evans PC Xu S. Nafld and Nash: etiology, targets and emerging therapies. Drug Discov Today. (2024) 29:103910. doi: 10.1016/j.drudis.2024.103910 38301798

[3] Zhou J Zhou F Wang W Zhang XJ Ji YX Zhang P . Epidemiological features of Nafld from 1999 to 2018 in China. Hepatology. (2020) 71:1851–64. doi: 10.1002/hep.31150, 32012320

[4] Wong VW Ekstedt M Wong GL Hagström H. Changing epidemiology, global trends and implications for outcomes of Nafld. J Hepatol. (2023) 79:842–52. doi: 10.1016/j.jhep.2023.04.036, 37169151

[5] Friedman SL Neuschwander-Tetri BA Rinella M Sanyal AJ. Mechanisms of Nafld development and therapeutic strategies. Nat Med. (2018) 24:908–22. doi: 10.1038/s41591-018-0104-9, 29967350 PMC6553468

[6] Paik JM Henry L Younossi Y Ong J Alqahtani S Younossi ZM. The burden of nonalcoholic fatty liver disease (Nafld) is rapidly growing in every region of the world from 1990 to 2019. Hepatol Commun. (2023) 7:e0251. doi: 10.1097/hc9.0000000000000251 PMC10545420 37782469

[7] Ferguson D Finck BN. Emerging therapeutic approaches for the treatment of Nafld and type 2 diabetes mellitus. Nat Rev Endocrinol. (2021) 17:484–95. doi: 10.1038/s41574-021-00507-z, 34131333 PMC8570106

[8] European Association for the Study of the Liver (EASL) EAftSoDE, & European Association for the Study of Obesity (EASO). Easl-Easd-Easo clinical practice guidelines on the management of metabolic dysfunction-associated steatotic liver disease (Masld). J Hepatol. (2024) 81:492–542. doi: 10.1016/j.jhep.2024.04.031 38851997

[9] Harrison SA Allen AM Dubourg J Noureddin M Alkhouri N. Challenges and opportunities in Nash drug development. Nat Med. (2023) 29:562–73. doi: 10.1038/s41591-023-02242-6, 36894650

[10] Zheng L Li B Yuan A Bi S Puscher H Liu C . Tfeb activator Tanshinone Iia and derivatives derived from *Salvia Miltiorrhiza* Bge. Attenuate hepatic steatosis and insulin resistance. J Ethnopharmacol. (2024) 335:118662. doi: 10.1016/j.jep.2024.118662, 39117022

[11] Dong H Zhao Y Teng H Jiang T Yue Y Zhang S . *Pueraria Lobata* antioxidant extract ameliorates non-alcoholic fatty liver by altering hepatic fat accumulation and oxidative stress. J Ethnopharmacol. (2024) 333:118468. doi: 10.1016/j.jep.2024.118468, 38906339

[12] Luo H Wu H Wang L Xiao S Lu Y Liu C . Hepatoprotective effects of Cassiae semen on mice with non-alcoholic fatty liver disease based on gut microbiota. Commun Biol. (2021) 4:1357. doi: 10.1038/s42003-021-02883-8, 34862475 PMC8642482

[13] Lei S Zhao S Huang X Feng Y Li Z Chen L . Chaihu Shugan powder alleviates liver inflammation and hepatic steatosis in Nafld mice: a network pharmacology study and in vivo experimental validation. Front Pharmacol. (2022) 13:967623. doi: 10.3389/fphar.2022.967623, 36172180 PMC9512055

[14] Yang R Jiang D Xu H Yang H Feng L Wu Q . Network pharmacology and molecular docking integrated with molecular dynamics simulations investigate the pharmacological mechanism of Yinchenhao decoction in the treatment of non-alcoholic fatty liver disease. Curr Comput Aided Drug Des. (2025). 21:721–37. doi: 10.2174/0115734099305489240702075128 38994616

[15] Dai X Feng J Chen Y Huang S Shi X Liu X . Traditional Chinese medicine in nonalcoholic fatty liver disease: molecular insights and therapeutic perspectives. Chin Med. (2021) 16:68. doi: 10.1186/s13020-021-00469-4, 34344394 PMC8330116

[16] Liu Y Fan Y Liu J Liu X Li X Hu J. Application and mechanism of Chinese herb medicine in the treatment of non-alcoholic fatty liver disease. Front Pharmacol. (2024) 15:1499602. doi: 10.3389/fphar.2024.1499602, 39605910 PMC11598537

[17] Zhang WY Wang MH Xie C. Potential of traditional Chinese medicine in the treatment of nonalcoholic fatty liver disease: a promising future. World J Gastroenterol. (2024) 30:4597–601. doi: 10.3748/wjg.v30.i43.4597, 39575403 PMC11572638

[18] Wu F Gao J Kang J Wang X Niu Q Liu J . Knowledge mapping of exosomes in autoimmune diseases: a bibliometric analysis (2002-2021). Front Immunol. (2022) 13:939433. doi: 10.3389/fimmu.2022.939433, 35935932 PMC9353180

[19] Zeng N Sun JX Liu CQ Xu JZ An Y Xu MY . Knowledge mapping of application of image-guided surgery in prostate Cancer: a bibliometric analysis (2013-2023). Int J Surg. (2024) 110:2992–3007. doi: 10.1097/js9.0000000000001232, 38445538 PMC11093506

[20] van Eck NJ Waltman L. Software survey: Vosviewer, a computer program for bibliometric mapping. Scientometrics. (2010) 84:523–38. doi: 10.1007/s11192-009-0146-3, 20585380 PMC2883932

[21] Chen C. Searching for intellectual turning points: progressive knowledge domain visualization. Proc Natl Acad Sci USA. (2004) 101 Suppl 1:5303–10. doi: 10.1073/pnas.0307513100, 14724295 PMC387312

[22] Aria M Cuccurullo C. Bibliometrix: an R-tool for comprehensive science mapping analysis. J Informetr. (2017) 11:959–75. doi: 10.1016/j.joi.2017.08.007

[23] Jiang S Liu Y Zheng H Zhang L Zhao H Sang X . Evolutionary patterns and research Frontiers in neoadjuvant immunotherapy: a bibliometric analysis. Int J Surg. (2023) 109:2774–83. doi: 10.1097/js9.0000000000000492, 37216225 PMC10498839

[24] Ai S Li Y Zheng H Zhang M Tao J Liu W . Collision of herbal medicine and nanotechnology: a bibliometric analysis of herbal nanoparticles from 2004 to 2023. J Nanobiotechnology. (2024) 22:140. doi: 10.1186/s12951-024-02426-3, 38556857 PMC10983666

[25] AlRyalat SAS Malkawi LW Momani SM. Comparing bibliometric analysis using Pubmed, Scopus, and web of science databases. J Vis Exp. (2019) 152:58494. doi: 10.3791/58494 31710021

[26] Powell EE Wong VW Rinella M. Non-alcoholic fatty liver disease. Lancet. (2021) 397:2212–24. doi: 10.1016/s0140-6736(20)32511-3, 33894145

[27] Chen M Xie Y Gong S Wang Y Yu H Zhou T . Traditional Chinese medicine in the treatment of nonalcoholic steatohepatitis. Pharmacol Res. (2021) 172:105849. doi: 10.1016/j.phrs.2021.105849, 34450307

[28] Han R Qiu H Zhong J Zheng N Li B Hong Y . Si Miao formula attenuates non-alcoholic fatty liver disease by modulating hepatic lipid metabolism and gut microbiota. Phytomedicine. (2021) 85:153544. doi: 10.1016/j.phymed.2021.153544, 33773192

[29] Wang Q Ou Y Hu G Wen C Yue S Chen C . Naringenin attenuates non-alcoholic fatty liver disease by down-regulating the Nlrp3/Nf-Κb pathway in mice. Br J Pharmacol. (2020) 177:1806–21. doi: 10.1111/bph.14938, 31758699 PMC7070172

[30] Hui D Liu L Azami NLB Song J Huang Y Xu W . The spleen-strengthening and liver-draining herbal formula treatment of non-alcoholic fatty liver disease by regulation of intestinal Flora in clinical trial. Front Endocrinol (Lausanne). (2022) 13:1107071. doi: 10.3389/fendo.2022.1107071, 36743913 PMC9892935

[31] Zhang SJ Chen ZX Jiang KP Cheng YH Gu YL. The effect of Quyuhuatantongluo decoction on the non-alcoholic steatohepatitis. Complement Ther Med. (2008) 16:192–8. doi: 10.1016/j.ctim.2007.08.004, 18638709

[32] Koperska A Moszak M Seraszek-Jaros A Bogdanski P Szulinska M. Does Berberine impact anthropometric, hepatic, and metabolic parameters in patients with metabolic dysfunction-associated fatty liver disease? Randomized, double-blind placebo-controlled trial. J Physiol Pharmacol. (2024) 75:291–302. doi: 10.26402/jpp.2024.3.06, 39042390

[33] Wang WY Zhou H Wang YF Sang BS Liu L. Current policies and measures on the development of traditional Chinese medicine in China. Pharmacol Res. (2021) 163:105187. doi: 10.1016/j.phrs.2020.105187, 32916255 PMC7480280

[34] Leung C Rivera L Furness JB Angus PW. The role of the gut microbiota in NAFLD. Nat Rev Gastroenterol Hepatol. (2016) 13:412–25. doi: 10.1038/nrgastro.2016.85 27273168

[35] Guo J Shi A Sun Y Zhang S Feng X Chen Y . Network pharmacology and experimental validation of the effects of Shenling Baizhu san, Quzhi Ruangan Fang and Gexia Zhuyu Tang on the intestinal flora of rats with NAFLD. Diabetes Metab Syndr Obes. (2025) 18:1165–94. doi: 10.2147/dmso.S507039, 40260263 PMC12011051

[36] Chen J Vitetta L. Gut microbiota metabolites in Nafld pathogenesis and therapeutic implications. Int J Mol Sci. (2020) 21:5214. doi: 10.3390/ijms21155214, 32717871 PMC7432372

[37] Xu L Xu K Xiong P Zhong C Zhang X Gao R . Zhuyu pill alleviates nonalcoholic fatty liver disease by regulating bile acid metabolism through the gut-liver Axis. ACS Omega. (2023) 8:29033–45. doi: 10.1021/acsomega.3c01955, 37599938 PMC10433349

[38] Zeng XY Zhang X Su H Gou HY Lau HCH Hu XX . Pien Tze Huang protects against non-alcoholic steatohepatitis by modulating the gut microbiota and metabolites in mice. Engineering. (2024) 35:257–69. doi: 10.1016/j.eng.2022.10.010

[39] Zou J Xiang Q Tan D Shi L Liu X Wu Y . Zuogui-Jiangtang-Qinggan-Fang alleviates high-fat diet-induced type 2 diabetes mellitus with non-alcoholic fatty liver disease by modulating gut microbiome-metabolites-short chain fatty acid composition. Biomed Pharmacother. (2023) 157:114002. doi: 10.1016/j.biopha.2022.114002, 36410120

[40] Chen L Zhang L Liu S Hua H Zhang L Liu B . Ling-Gui-Zhu-Gan decoction ameliorates nonalcoholic fatty liver disease via modulating the gut microbiota. Microbiol Spectrum. (2024) 12:e0197923. doi: 10.1128/spectrum.01979-23 PMC11237417 38647315

[41] Cai Y Fang L Chen F Zhong P Zheng X Xing H . Targeting Ampk related signaling pathways: a feasible approach for natural herbal medicines to intervene non-alcoholic fatty liver disease. J Pharm Anal. (2025) 15:101052. doi: 10.1016/j.jpha.2024.101052, 40034684 PMC11873010

[42] Lei Y Wang L Song L Han J Ma H Luo H . Tiaogan Jiejiu Tongluo formula alleviates hepatic steatosis in NAFLD mice by regulating AMPK signaling pathway. J Pharm Pharmacol. (2025) 77:492–500. doi: 10.1093/jpp/rgaf005, 39951113

[43] Zhu J Guo J Liu Z Liu J Yuan A Chen H . Salvianolic acid a attenuates non-alcoholic fatty liver disease by regulating the AMPK-Igfbp1 pathway. Chem Biol Interact. (2024) 400:111162. doi: 10.1016/j.cbi.2024.111162, 39047806

[44] Wang MY Zhang SS An MF Xia YF Fan MS Sun ZR . Neferine ameliorates nonalcoholic steatohepatitis through regulating AMPK pathway. Phytomedicine. (2023) 114:154798. doi: 10.1016/j.phymed.2023.154798, 37031639

[45] Wang X Abu Bakar MH Liqun S Kassim MA Shariff KA Karunakaran T. Targeting metabolic diseases with Celastrol: a comprehensive review of anti-inflammatory mechanisms and therapeutic potential. J Ethnopharmacol. (2025) 344:119560. doi: 10.1016/j.jep.2025.119560, 40015541

[46] Tian J Cai M Jin S Chen Q Xu J Guo Q . Jianpi-Qinghua formula attenuates nonalcoholic fatty liver disease by regulating the AMPK/Sirt1/Nf-Κb pathway in high-fat-diet-fed C57bl/6 mice. Pharm Biol. (2023) 61:647–56. doi: 10.1080/13880209.2023.2188549, 37038833 PMC10101667

[47] Fu K Dai S Ma C Zhang YF Zhang SL Wang C . Lignans are the main active components of Schisandrae Chinensis Fructus for liver disease treatment: a review. Food Sci Human Wellness. (2024) 13:2425–44. doi: 10.26599/fshw.2022.9250200

[48] Schoeler M Caesar R. Dietary lipids, gut microbiota and lipid metabolism. Rev Endocr Metab Disord. (2019) 20:461–72. doi: 10.1007/s11154-019-09512-0, 31707624 PMC6938793

[49] Zhao M Chu J Feng S Guo C Xue B He K . Immunological mechanisms of inflammatory diseases caused by gut microbiota Dysbiosis: a review. Biomed Pharmacother. (2023) 164:114985. doi: 10.1016/j.biopha.2023.114985, 37311282

[50] Glass CK Olefsky JM. Inflammation and lipid signaling in the etiology of insulin resistance. Cell Metab. (2012) 15:635–45. doi: 10.1016/j.cmet.2012.04.001, 22560216 PMC4156155

[51] Zeng MY Inohara N Nuñez G. Mechanisms of inflammation-driven bacterial dysbiosis in the gut. Mucosal Immunol. (2017) 10:18–26. doi: 10.1038/mi.2016.75, 27554295 PMC5788567

[52] Feng X Sureda A Jafari S Memariani Z Tewari D Annunziata G . Berberine in cardiovascular and metabolic diseases: from mechanisms to therapeutics. Theranostics. (2019) 9:1923–51. doi: 10.7150/thno.30787, 31037148 PMC6485276

[53] Xu X Yi H Wu J Kuang T Zhang J Li Q . Therapeutic effect of berberine on metabolic diseases: both pharmacological data and clinical evidence. Biomed Pharmacother. (2021) 133:110984. doi: 10.1016/j.biopha.2020.110984, 33186794

[54] Yang S Cao SJ Li CY Zhang Q Zhang BL Qiu F . Berberine directly targets Akr1b10 protein to modulate lipid and glucose metabolism disorders in Nafld. J Ethnopharmacol. (2024) 332:118354. doi: 10.1016/j.jep.2024.118354, 38762210

[55] Wang Y Tai YL Zhao D Zhang Y Yan J Kakiyama G . Berberine prevents disease progression of nonalcoholic steatohepatitis through modulating multiple pathways. Cells. (2021) 10:210. doi: 10.3390/cells10020210, 33494295 PMC7912096

[56] Zhu LR Li SS Zheng WQ Ni WJ Cai M Liu HP. Targeted modulation of gut microbiota by traditional Chinese medicine and natural products for liver disease therapy. Front Immunol. (2023) 14:1086078. doi: 10.3389/fimmu.2023.1086078, 36817459 PMC9933143

[57] Nie Q Li M Huang C Yuan Y Liang Q Ma X . The clinical efficacy and safety of Berberine in the treatment of non-alcoholic fatty liver disease: a Meta-analysis and systematic review. J Transl Med. (2024) 22:225. doi: 10.1186/s12967-024-05011-2, 38429794 PMC10908013

[58] Yan HM Xia MF Wang Y Chang XX Yao XZ Rao SX . Efficacy of Berberine in patients with non-alcoholic fatty liver disease. PLoS One. (2015) 10:e0134172. doi: 10.1371/journal.pone.0134172, 26252777 PMC4529214

[59] Harrison SA Gunn N Neff GW Kohli A Liu L Flyer A . A phase 2, proof of concept, randomised controlled trial of Berberine Ursodeoxycholate in patients with presumed non-alcoholic steatohepatitis and type 2 diabetes. Nat Commun. (2021) 12:5503. doi: 10.1038/s41467-021-25701-5, 34535644 PMC8448729

[60] Ma X Yu X Li R Cui J Yu H Ren L . Berberine-Silybin salt achieves improved anti-nonalcoholic fatty liver disease effect through regulating lipid metabolism. J Ethnopharmacol. (2024) 319:117238. doi: 10.1016/j.jep.2023.117238, 37774895

[61] Behl T Singh S Sharma N Zahoor I Albarrati A Albratty M . Expatiating the pharmacological and Nanotechnological aspects of the alkaloidal drug Berberine: current and future trends. Molecules. (2022) 27:3705. doi: 10.3390/molecules27123705, 35744831 PMC9229453

[62] Wang J Zang J Yu Y Liu Y Cao H Guo R . Lingguizhugan Oral solution alleviates Masld by regulating bile acids metabolism and the gut microbiota through activating Fxr/Tgr5 signaling pathways. Front Pharmacol. (2024) 15:1426049. doi: 10.3389/fphar.2024.1426049, 39211777 PMC11358101

[63] Yang L Lin W Nugent CA Hao S Song H Liu T . Lingguizhugan decoction protects against high-fat-diet-induced nonalcoholic fatty liver disease by alleviating oxidative stress and activating cholesterol secretion. Int J Genomics. (2017) 2017:2790864. doi: 10.1155/2017/2790864, 29464180 PMC5804362

[64] Cao L Xu E Zheng R Zhangchen Z Zhong R Huang F . Traditional Chinese medicine Lingguizhugan decoction ameliorate Hfd-induced hepatic-lipid deposition in mice by inhibiting sting-mediated inflammation in macrophages. Chin Med. (2022) 17:7. doi: 10.1186/s13020-021-00559-3, 34983596 PMC8728979

[65] Liu MT Huang YJ Zhang TY Tan LB Lu XF Qin J. Lingguizhugan decoction attenuates diet-induced obesity and hepatosteatosis via gut microbiota. World J Gastroenterol. (2019) 25:3590–606. doi: 10.3748/wjg.v25.i27.3590, 31367159 PMC6658390

[66] Dai L Xu J Liu B Dang Y Wang R Zhuang L . Lingguizhugan decoction, a Chinese herbal formula, improves insulin resistance in overweight/obese subjects with non-alcoholic fatty liver disease: a translational approach. Front Med. (2022) 16:745–59. doi: 10.1007/s11684-021-0880-3, 35471471

[67] Zhang P Zhang D Zhou W Wang L Wang B Zhang T . Network pharmacology: towards the artificial intelligence-based precision traditional Chinese medicine. Brief Bioinform. (2023) 25:bbad518. doi: 10.1093/bib/bbad518, 38197310 PMC10777171

[68] Zhang M Yuan Y Zhou W Qin Y Xu K Men J . Network pharmacology analysis of Chaihu Lizhong Tang treating non-alcoholic fatty liver disease. Comput Biol Chem. (2020) 86:107248. doi: 10.1016/j.compbiolchem.2020.107248, 32208163

[69] Yu C Han D Yu J Zhu R Zhu C Wang F . Exploration of potential targets and mechanisms of naringenin in the treatment of nonalcoholic fatty liver disease through network pharmacology. Medicine (Baltimore). (2023) 102:e35460. doi: 10.1097/md.0000000000035460 37861538 PMC10589567

[70] Pinzi L Rastelli G. Molecular docking: shifting paradigms in drug discovery. Int J Mol Sci. (2019) 20:4331. doi: 10.3390/ijms20184331, 31487867 PMC6769923

[71] Liu Z Shang Q Cheng J He Q Liu Y Li H . Mechanistic study of a triterpenoid-enriched fraction derived from Cynomorium Songaricum against Nafld: an integrative elucidation. Phytomedicine. (2025) 142:156782. doi: 10.1016/j.phymed.2025.156782 40318532

[72] Noor F Asif M Ashfaq UA Qasim M Tahir Ul Qamar M. Machine learning for synergistic network pharmacology: a comprehensive overview. Brief Bioinform. (2023) 24:bbad120. doi: 10.1093/bib/bbad120 37031957

