# Supplementary material for: Mapping the knowledge domain: a bibliometric analysis of global research on traditional Chinese medicine for non-alcoholic fatty liver disease (2000–2024)
Source: Front Med (Lausanne). 2026 Mar 20;13:1744929. doi: 10.3389/fmed.2026.1744929 (PMC13046715; doi:10.3389/fmed.2026.1744929)
Supplement: Supplementary file 1 [file Table_1.docx]

**Table S1**. Top 25 References with Strong Citation Bursts in the Field of TCM in NAFLD

| Rank | Strength | Title | Total Citations | Article type |
| --- | --- | --- | --- | --- |
| 1 | 15.06 | Global Epidemiology of Nonalcoholic Fatty Liver Disease-Meta-Analytic Assessment of Prevalence, Incidence, and Outcomes | 7694 | Review |
| 2 | 9.74 | Global burden of NAFLD and NASH: trends, predictions, risk factors and prevention | 4731 | Review |
| 3 | 9.72 | Mechanisms of NAFLD development and therapeutic strategies | 3746 | Review |
| 4 | 8.25 | Non-alcoholic fatty liver disease | 2374 | Review |
| 5 | 7.08 | [The multiple-hit pathogenesis of non-alcoholic fatty liver disease (NAFLD)](https://www.webofscience.com/wos/alldb/full-record/WOS:000379889400004) | 2791 | Review |
| 6 | 6.6 | NAFLD: A multisystem disease | 2716 | Review |
| 7 | 6.24 | [Traditional Chinese medicine in the treatment of nonalcoholic steatohepatitis](https://www.webofscience.com/wos/alldb/full-record/WOS:000696677900041) | 69 | Review |
| 8 | 5.79 | The diagnosis and management of nonalcoholic fatty liver disease: Practice guidance from the American Association for the Study of Liver Diseases | 5123 | Review |
| 9 | 5.57 | Herbal medicines and nonalcoholic fatty liver disease | 131 | Review |
| 10 | 5.2 | [New trends on obesity and NAFLD in Asia](https://www.webofscience.com/wos/alldb/full-record/WOS:000410775900022) | 1012 | Review |
| 11 | 5.13 | Traditional Chinese Medicine in nonalcoholic fatty liver disease: molecular insights and therapeutic perspectives | 88 | Review |
| 12 | 5.11 | Protective effect of quercetin on high-fat diet-induced non-alcoholic fatty liver disease in mice is mediated by modulating intestinal microbiota imbalance and related gut-liver axis activation | 490 | Original Research |
| 13 | 5.04 | Nonalcoholic Fatty Liver Disease A Systematic Review | 2166 | Review |
| 14 | 4.99 | Regulatory effect of a Chinese herbal medicine formula on non-alcoholic fatty liver disease | 89 | Original Research |
| 15 | 4.65 | Cause, Pathogenesis, and Treatment of Nonalcoholic Steatohepatitis | 1186 | Review |
| 16 | 4.55 | Global Perspectives on Nonalcoholic Fatty Liver Disease and Nonalcoholic Steatohepatitis | 1671 | Review |
| 17 | 4.5 | Human Fatty Liver Disease: Old Questions and New Insights | 2052 | Review |
| 18 | 4.41 | Si Miao Formula attenuates non-alcoholic fatty liver disease by modulating hepatic lipid metabolism and gut microbiota | 67 | Original Research |
| 19 | 4.4 | Nonalcoholic Fatty Liver Disease: Pathogenesis and Treatment in Traditional Chinese Medicine and Western Medicine | 52 | Review |
| 20 | 4.32 | Non-alcoholic fatty liver disease (NAFLD) - pathogenesis, classification, and effect on drug metabolizing enzymes and transporters | 623 | Review |
| 21 | 4.31 | The role of the gut microbiota in NAFLD | 922 | Review |
| 22 | 4.11 | Current and future pharmacological therapies for NAFLD/NASH | 630 | Review |
| 23 | 4.06 | Unexpected Rapid Increase in the Burden of NAFLD in China From 2008 to 2018: A Systematic Review and Meta-Analysis | 591 | Review |
| 24 | 4.02 | Modeling NAFLD disease burden in China, France, Germany, Italy, Japan, Spain, United Kingdom, and United States for the period 2016-2030 | 1740 | Review |
| 25 | 3.96 | Naringenin attenuates non-alcoholic fatty liver disease by down-regulating the NLRP3/NF-κB pathway in mice | 290 | Original Research |

**Table S2.** Summary of Clinical Trials on TCM in NAFLD

| No. | Year | Title | Intervention | Control Group | Sample Size | Conclusion |
| --- | --- | --- | --- | --- | --- | --- |
| 1 | 2008 | The effect of QuYuHuaTanTongLuo Decoction on the non-alcoholic steatohepatitis | QuYuHuaTanTongLuo Decoction(QYHTTLD) | Ursodeoxycholic acid (UDCA) | 69 | QYHTTLD significantly improved lipid profiles, inflammatory markers, oxidative stress indices, and liver ultrasound findings. Compared to UDCA, QYHTTLD showed greater improvement in symptom scores and biochemical parameters. |
| 2 | 2015 | Efficacy of Berberine in Patients with Non-Alcoholic Fatty Liver Disease | Berberine (BBR) (plus Lifestyle Intervention - LSI) | LSI alone2)Pioglitazone(PGZ)(plus LSI) | 184 | Compared to LSI alone, BBR significantly reduced liver fat (HFC), body weight, HOMA-IR, and lipids. BBR was also more effective than Pioglitazone in reducing weight and improving the lipid profile. Adverse events were mild. |
| 3 | 2018 | Clinical effect of the extract of TCM Fructus akebiae combined with ursodeoxycholic acid on nonalcoholic fatty liver disease | Fructus akebiae extract (FAE) | Ursodeoxycholic acid (UDCA) | 180 | The FAE + UDCA group showed significantly higher comprehensive clinical efficacy than the UDCA-only group. The FAE add-on also significantly improved clinical symptoms and biochemical indicators (ALT, AST, TG, TC). |
| 4 | 2021 | A phase 2, proof of concept, randomised controlled trial of berberine ursodeoxycholate in patients with presumed non-alcoholic steatohepatitis and type 2 diabetes | Berberine ursodeoxycholate(HTD1801) | Placebo | 100 | The HTD1801 group had a significantly greater reduction in liver fat content vs. placebo. This dose also significantly improved glycemic control, reduced liver enzymes, and caused significant weight loss. It was relatively well tolerated. |
| 5 | 2022 | Danshao Shugan Granule therapy for non-alcoholic fatty liver disease | Danshao Shugan Granules (DSSG) | Rosiglitazone (Ros) & Silibinin (Sil) (Positive drug controls) | 260 | DSSG was effective for treating NAFLD patients. Compared to active controls, DSSG had the highest effects on B-ultrasonography improvements and reductions of TC, TG, AST, and GGT. Rosiglitazone led to the highest FPG reductions, and Silibinin led to the highest ALT reductions. |
| 6 | 2023 | Lingguizhugan Decoction, a Chinese herbal formula, improves insulin resistance in overweight/obese subjects with non-alcoholic fatty liver disease: a translational approach | Lingguizhugan Decoction (LGZG) | Placebo | 243 | Low-dose LGZG significantly improved insulin resistance (HOMA-IR) in overweight/obese NAFLD subjects compared to placebo. No superior effect was observed in lean subjects. |
| 7 | 2022 | Effects of Shenxiang Suhe Pill on coronary heart disease complicated with nonalcoholic fatty liver disease: A case-control study | Shenxiang Suhe Pill | Conventional western medicines | 56 | Compared to control, the Shenxiang Suhe Pill (add-on) group showed a significant decrease in liver fat (CAP value), NAFLD severity, TG, LDH, and hs-CRP. HDL also improved. No significant changes were observed in ALT or AST. |
| 8 | 2023 | The spleen-strengthening and liver-draining herbal formula treatment of non-alcoholic fatty liver disease by regulation of intestinal flora in clinical trial | Spleen-strengthening and liver-draining formula (SLF) | Lifestyle adjustments (diet and exercise) | 88 | Superior to lifestyle adjustments alone in improving liver function, CAP (fat), LSM (stiffness), lipids, and HOMA-IR.. Efficacy linked to gut flora modulation (e.g., Coprococcus, Ruminococcus). Showed good security |
| 9 | 2024 | Efficacy and safety of Qushi Huayu, a traditional Chinese medicine, in patients with nonalcoholic fatty liver disease in a randomized controlled trial | Qushi Huayu (QSHY) granules | Dangfei Liganning capsule (DFLG) | 246 | Superior to DFLG in improving serum ALT, AST, and FIB-4 score. Efficacy was linked to modulation of gut dysbiosis (e.g., Escherichia-Shigella and Bacteroides) |
| 10 | 2024 | Does berberine impact anthropometric, hepatic, and metabolic parameters in patients with metabolic dysfunction-associated fatty liver disease? Randomized, double-blind placebo-controlled trial | Berberine (BBR) | Placebo | 70 | Compared to placebo, BBR significantly decreased ALT, the de Ritis ratio, and total cholesterol (TC) after 12 weeks. However, no significant differences were found in other lipid or glucose parameters. |
